# Efficacy of supraspinatus tendon repair using mesenchymal stem cells along with a collagen I scaffold

**DOI:** 10.1186/s13018-015-0269-6

**Published:** 2015-08-14

**Authors:** Pilar Tornero-Esteban, José Antonio Hoyas, Esther Villafuertes, Cruz Rodríguez-Bobada, Yamila López-Gordillo, Francisco J. Rojo, Gustavo V. Guinea, Anna Paleczny, Yaiza Lópiz-Morales, Luis Rodriguez-Rodriguez, Fernando Marco, Benjamín Fernández-Gutiérrez

**Affiliations:** UGC de Reumatología, Hospital Clínico San Carlos, Instituto de Investigación Sanitaria del Hospital Clínico San Carlos (IdISSC), Madrid, Spain; Dpto de Medicina y Cirugía Experimentales, Hospital Clínico San Carlos, Instituto de Investigación Sanitaria del Hospital Clínico San Carlos (IdISSC), Madrid, Spain; Dpto de Anatomía y Embriología Humana I, Facultad de Medicina Universidad, Complutense de Madrid, Madrid, Spain; Dpto de Ciencia de Materiales, Universidad Politécnica de Madrid, Madrid, Spain; Centro de Tecnología Biomédica, Universidad Politécnica de Madrid, Pozuelo de Alarcón, Madrid, Spain; Servicio de Cirugía Ortopédica y Traumatología, Hospital Clínico San Carlos, Instituto de Investigación Sanitaria del Hospital Clínico San Carlos (IdISSC), Madrid, Spain

**Keywords:** Mesenchymal stem cells (MSCs), Animal model, Rotator cuff

## Abstract

**Objectives:**

Our main objective was to biologically improve rotator cuff healing in an elderly rat model using mesenchymal stem cells (MSCs) in combination with a collagen membrane and compared against other current techniques.

**Methods:**

A chronic rotator cuff tear injury model was developed by unilaterally detaching the supraspinatus (SP) tendons of Sprague-Dawley rats. At 1 month postinjury, the tears were repaired using one of the following techniques: (a) classical surgery using sutures (*n* = 12), (b) type I collagen membranes (*n* = 15), and (c) type I collagen membranes + 1 × 106 allogeneic MSCs (*n* = 14). Lesion restoration was evaluated at 1, 2, and 3 months postinjury based on biomechanical criteria. Continuous variables were described using mean and standard deviation (SD). To analyse the effect of the different surgical treatments in the repaired tendons’ biomechanical capabilities (maximum load, stiffness, and deformity), a two-way ANOVA model was used, introducing an interaction between such factor and time (1, 2, and 3 months postinjury).

**Results:**

With regard to maximum load, we observed an almost significant interaction between treatment and time (*F* = 2.62, *df* = 4, *p* = 0.053). When we analysed how this biomechanical capability changed with time for each treatment, we observed that repair with OrthADAPT and MSCs was associated with a significant increase in maximum load (*p* = 0.04) between months 1 and 3. On the other hand, when we compared the different treatments among themselves at different time points, we observed that the repair with OrthADAPT and MSCs has associated with a significant higher maximum load, when compared with the use of suture, but only at 3 months (*p* = 0.014). With regard to stiffness and deformity, no significant interaction was observed (*F* = 1.68, *df* = 4, *p* = 0.18; *F* = 0.40, *df* = 4, *p* = 0.81; respectively).

**Conclusions:**

The implantation of MSCs along with a collagen I scaffold into surgically created tendon defects is safe and effective. MSCs improved the tendon’s maximum load over time, indicating that MSCs could help facilitate the dynamic process of tendon repair.

## Background

The rotator cuff is formed by a group of muscles and tendons that stabilise the glenohumeral joint and enable the normal movement of the shoulder. Rotator cuff tears can occur due to traumatic injury, but most occur as a result of the gradual degeneration of the tendon. Consequently, elderly individuals are often affected in a chronic manner. Partial and/or full thickness tears in the rotator cuff tendons are common causes of pain and disability of the shoulder and can ultimately lead to osteoarthritis.

The surgical procedures that are typically used to treat these lesions, which use sutures to reattach the torn tendons to the bone, are unreliable, and lesion reoccurrence is very frequent reaching more than 50 % [1]. Due to the weakness of the fibres in the affected tendon, primarily the supraspinatus (SP) tendon, at its insertion site, it is very difficult to correctly reattach the tendon during surgery [2]. Clinical failure in degenerative lesions has been linked to preoperative factors such as age [3], reduced acromiohumeral distance [4], tear size [5–8], chronically inferior tissue quality and tendon retraction and to surgical factors such as high repair tension [9]. All of these factors have a marked effect on biological healing and remodelling, which eventually influence the clinical outcome. When full-thickness tears occur in the elderly, in whom the natural healing ability is greatly reduced, it is crucial to reduce the in vivo mechanical forces that work against tendon repair during postoperative healing and to enhance natural healing, thereby preventing tendon re-ruptures and eventually resulting in a better clinical outcome. Therefore, there is a need for new approaches that improve the healing process through mechanical re-enforcement and enhance natural healing, especially at the insertion site. In this context, different therapies in the field of tendon regeneration, such as the local injection of mesenchymal stem cells (MSCs) and growth factors or tissue engineering using bioactive biological membranes, have been tested in different animal models [10–16]. These approaches have been reported to improve tendon repair, although the complete regeneration of the tendon has never been achieved. Although the research in this field is intense, the majority of studies have focused on the treatment of traumatic injuries, especially in young animals, in which the muscle and tendon are not as degraded as the tissue in chronic lesions [17]. As a result of these differences, these animal studies report better outcomes than are likely to occur in humans. In the current study, we developed and validated a massive chronic rotator cuff tear rat model to explore different repair strategies. We compared three different strategies: a surgical procedure based on a modified Masson-Allen stitch using a suture, a surgical procedure using a commercially available biocomposite scaffold consisting of type I collagen that is available for human use and a surgical procedure using the type I collagen scaffold in combination with allogeneic MSCs.

## Materials and methods

### Study design

Sprague-Dawley rats (9 months old) were included in this study. The three therapeutic approaches were performed 1 month postdetachment of the SP tendon and are described as follows: (a) a surgical procedure based on a modified Masson-Allen stitch using a suture (6/0 prolene; *n* = 12), (b) a surgical procedure using a 0.5-mm-thick commercial type I collagen membrane (OrthADAPT® Bioimplant, Penta Biomedical® S.r.l., Verona, Italy; *n* = 15) and (c) a surgical procedure using the type I collagen membrane seeded with 1 × 106 MSCs (*n* = 14).

### Validation of experimental rat model of chronic rotator cuff tears

**Fig. 1:**
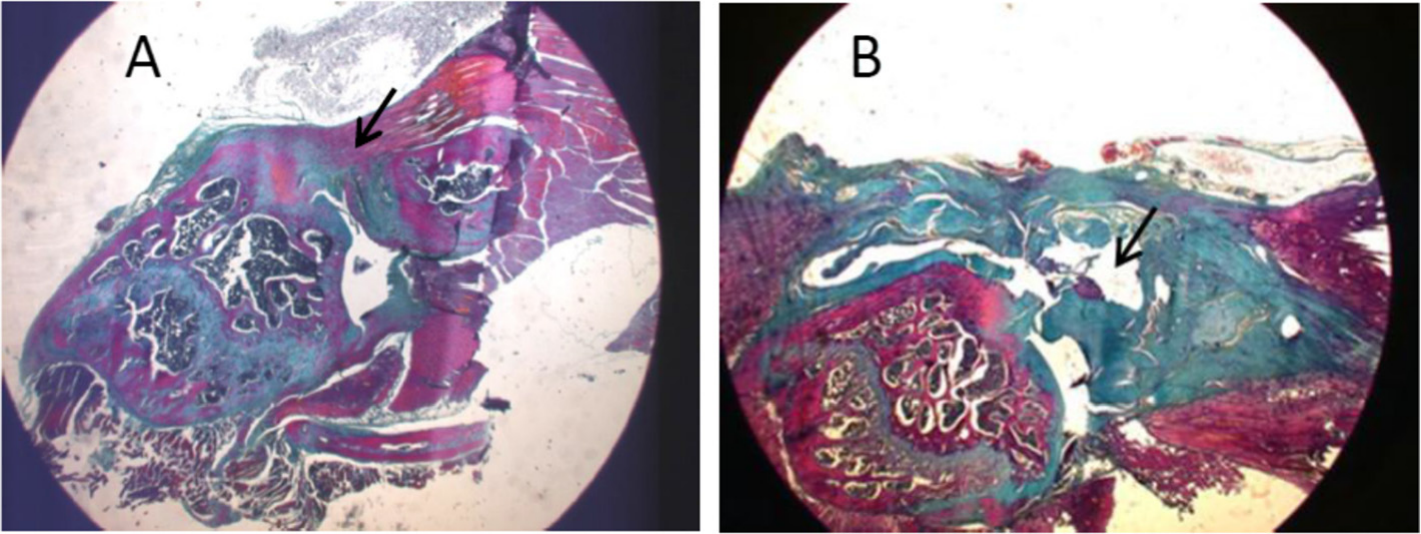
Representative image of established chronic supraspinatus lesion. *Black arrows* represent the tendon-bone interface. **a** Gap is clearly observed in **b** magnification ×125. **a** Normal tendon. **b** Injured tendon

To generate chronic rotator tears in the animal model, a tear was surgically created in one of the shoulders by sharply detaching the SP tendon from its insertion site. While adhering to aseptic operating room protocols, the deltoid muscle was subsequently detached sharply from the acromion. The SP was visualised and then exposed by external rotation of the humerus, and it was detached sharply at the bone insertion site. The end of the tendon was marked with a suture (5/0 prolene) with a long tail. The fibrocartilage was removed from the insertion site with a scalpel blade, and the SP tendon was allowed to freely retract. The overlying deltoid muscle and skin were then closed (Fig. 1). Rats were sacrificed at months 1 (*n* = 16), 2 (*n* = 13), and 3 (*n* = 12) postinjury, and specimens (humerus and scapula) were harvested. Recovery was evaluated through biomechanical evaluations. Animal care was performed in accordance with institution guidelines. The approval was given by the Ethics Committee of the Hospital Clinico San Carlos.

### Different approaches for the repair chronic rotator cuff tears in the rat model

One month after the injury, one of three different additional surgeries was performed to repair the tears: (a) the typical surgical procedure using a suture (6/0 prolene), (b) a surgical procedure using a 0.5-mm-thick commercial type I collagen membrane (OrthADAPT®Bioimplant, Penta Biomedical® S.r.l., Verona, Italy), and (c) a surgical procedure using a type I collagen membrane seeded with 1 × 106 MSCs. Scar tissue from the first surgery was removed before the second surgical procedure was performed. The rats were sacrificed at 1, 2, and 3 months postinjury, and specimens (humerus and scapula) were harvested. Recovery was evaluated at the time of sacrifice by biomechanical analyses.

### Cells and type I collagen membrane (OrthADAPT) preparation

MSCs were obtained from the femoral channel of age-matched rats (9 months old) prior to the study. The cells were placed in 100-mm dishes, and the cultures were maintained for 48 h under standard conditions. MSCs adhered to the plates and proliferated to form colonies. When the cultures reached confluence, the MSC colonies that adhered to the plate were detached using 0.25 % Trypsin-EDTA (Gibco BRL Life Technologies, Carlsbad, CA, USA). The cells were counted, and their viability was determined using the Trypan blue exclusion method. Aliquots containing 1 × 106 cells were suspended in 200 μl of saline. OrthADAPT membranes were placed on the cell suspension containing 1 × 10^6^ cells MSCs within 15 min before the event of surgical procedure. Previously, we have established that 5 min is the time the cells need in order to be adherent on the surface. No MSCs were observed in the remaining saline liquid.

### Biomechanical analysis

Specimens (humerus and scapula) were dissected and stored at −20 °C until analysis. For the mechanical testing, both sides of the tendon (humerus and scapula) were wrapped tightly with elastic rubber to prevent the sample from slipping out of the tube during the tensile test. For additional support, the rubber was fixed to the sample smear on both ends with the use of superglue. The tendons were wrapped in gauze and moistened with physiological serum to protect them. Two sets of tubes were prepared for the attachment of the samples to tensile machine grips: smaller tubes made of silicone (15 mm diameter) for the humerus and larger tubes made of polypropylene (25 mm diameter) for the scapula. One end of each tube was sealed with a piece of foil to prevent leakage of the resin. To prepare the resin, we mixed two types of glue (Araldite, Huntsman Advanced Materials, Basel, Switzerland) in equal proportions (50/50 %). Afterwards, the resin was poured into the smaller tube in which the humerus was placed, being careful to support the other end of the supraspinatus complex (scapula) to preserve the physiological angle between the humerus and scapula. We then prepared the resin that was poured into the larger tube, which contained the scapula, in the same manner. The specimens were tested parallel to the long axis of the SP tendon in their physiological position using an Instron 5866 electromechanical testing instrument. The velocity used during testing was 1.8 mm/min. The samples were loaded up to 10 N during the test, unloaded to the initial point and then loaded again until they reached the failure point. To estimate the stiffness of the tendons, the tensile strength machine registered the load [N] and displacement [mm]. The repaired stiffness was defined as the slope of the load divided by the displacement at the failure point. Failure was defined as the maximum value of the load needed to permanently damage the sample.

### Statistical analysis

Continuous variables were described using mean and standard deviation (SD). To analyse the effect of the different surgical treatments in the repaired tendons’ biomechanical capabilities (maximum load, stiffness and deformity), a two-way ANOVA model was used, introducing an interaction between such factor and time (1, 2 and 3 months postinjury). Simple effects analysis, adjusted by Sidak’s method, was used to study both the effects of time at each treatment level and the effects of treatment at each time. Effects size was calculated using Cohen’s *f*.

A priori sample size calculation was performed using G*Power (Heinrich-Heine-University Düsseldorf), assuming that the interaction between time (1 and 3 month) and the treatment (repair with OrthADAPT and MSCs) compared with the use of suture had a large effect size (Cohen’s *f* = 0.40), a two-tailed type I error of 0.05, and an 80 % power. Based on such values, we would need a total sample size of 52 animals. Also, a post hoc statistical power calculation was performed. Statistical analyses were performed using STATA v12.

## Results

### Macroscopic examinations of the specimens

**Fig. 2:**
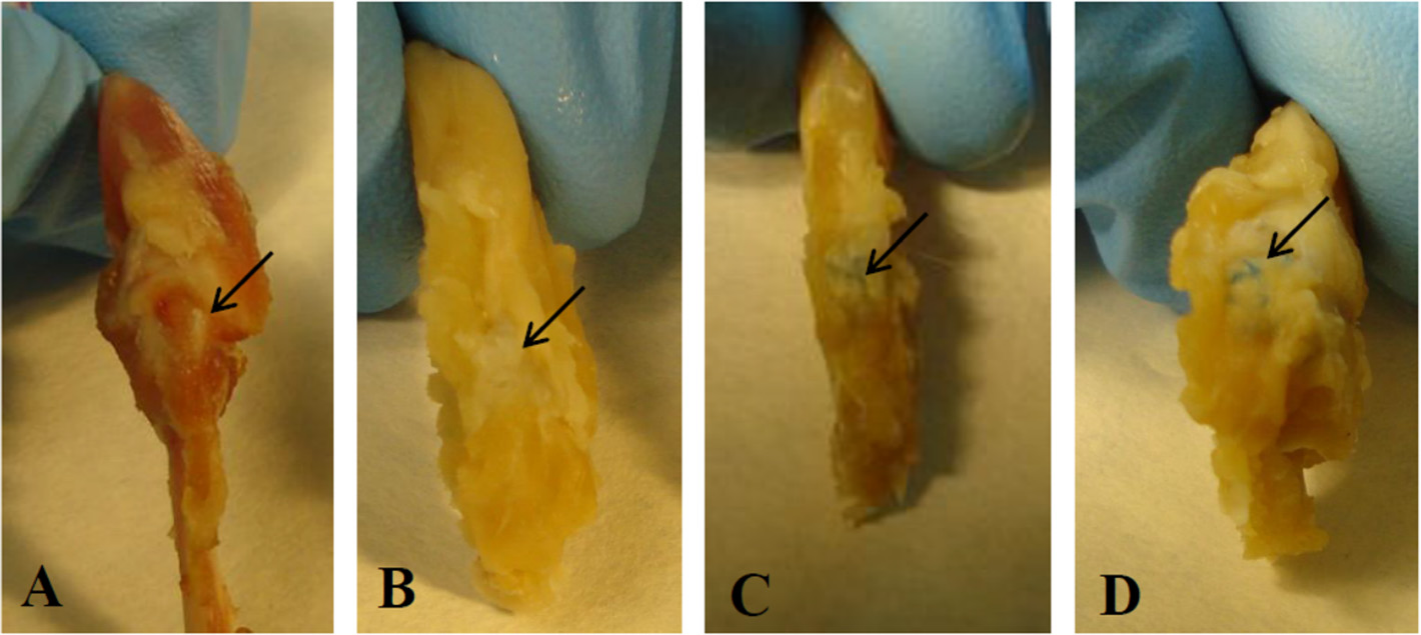
Macroscopic view of shoulders. **a** Normal shoulder. **b** Suture. **c** Suture plus collagen membrane. **d** Suture plus collagen membrane plus mesenchymal stem cells. *Black arrows* represent the tendon-bone interface

Gross examinations of the specimens at the time of dissection at 3 months after the repair treatment revealed scar tissue around the healed area with inflammation signs in all the study groups. Nevertheless, the signs of inflammation were seen to be more prominent in animals treated with suture or collagen membrane in contrast with rats treated with MSCs. The treatment with MSCs provided a clearer view of the repair area indicative of a minor scar inflammatory condition (Fig. 2).

### Different strategies for the repair of injured SP tendons

Biomechanical tests of the repaired SP tendons are summarised in Table 1. With regard to maximum load, we observed an almost significant interaction between treatment and time (*F* = 2.62, *df* = 4, *p* = 0.053). Effect size for such interaction was large (Cohen’s *f* = 0.41), and therefore, we decided to perform simple effects analysis, despite this lack of significance, to better characterise the relationship between both factors and their effect in maximum load. When we analysed how this biomechanical capability changed with time for each treatment, we observed that repair with OrthADAPT and MSCs was associated with a significant increase in maximum load (*p* = 0.04), between months 1 and 3 (Table 2). On the other hand, when we compared the different treatments among themselves at different time points, we observed that the repair with OrthADAPT and MSCs has associated with a significant higher maximum load, when compared with the use of suture, but only at 3 months (*p* = 0.014) (Table 3). With regard to stiffness and deformity, no significant interaction was observed (*F* = 1.68, *df* = 4, *p* = 0.18; *F* = 0.40, *df* = 4, *p* = 0.81; respectively).

**Table 1:** Biomechanical characteristics

		*n*	Suture mean (SD)	*n*	OrthADAPT mean (SD)	*n*	OrthADAPT + MSCs mean (SD)
Max. load							
	1	4	34.84 (6.96)	6	24.16 (15.81)	6	25.27 (9.96)
Time (months)	2	4	29.77 (10.81)	4	32,59 (15.65)	5	27.86 (11.1)
	3	4	20.63 (11.69)	5	31.43 (6.97)	3	45.01 (1.84)
Deformation							
	1	4	7.5 (2.3)	6	6.13 (2.72)	6	10.08 (9.37)
Time (months)	2	4	10.08 (2.24)	4	8.92 (2.97)	5	7.92 (5.21)
	3	3	8.01 (3.42)	5	7.82 (7.17)	4	7.9 (3.42)
Stiffness							
	1	4	4.83 (0.94)	6	3.03 (1.66)	6	2.58 (1.26)
Time (months)	2	4	3.03 (1.02)	4	5.33 (4.13)	5	2.82 (1.12)
	3	3	2.63 (1.06)	5	5.56 (4.11)	4	4.45 (0.57)

*Max. load* maximum load, *SD* standard deviation, *MSCs* mesenchymal stem cells

**Table 2:** Biomechanical characteristics

	Suture	OrthADAPT	OrthADAPT + MSCs
2 vs. 1 month	0.89	0.57	0.97
3 vs. 1 month	0.21	0.64	0.04^*^
3 vs. 2 months	0.58	0.99	0.11

Maximum load by time

*MSCs* mesenchymal stem cells

^*^ *p* < 0.05

**Table 3:** Biomechanical characteristics

	1 month	2 months	3 months
OrthADAPT vs. suture	0.37	0.98	0.39
OrthADAPT + MSCs vs. suture	0.47	0.99	0.014^*^
OrthADAPT + MSCs vs. OrthADAPT	0.99	0.90	0.27

Maximum load by different approaches for the repair

*MSC*s mesenchymal stem cells

^*^ *p* < 0.05

In the post hoc statistical power calculation, based on the observed results, our study achieved a 70 % power.

## Discussion

Despite the great advances in the surgical procedures used to treat rotator cuff tears, the frequency of re-rupture is still unacceptable for large injuries. Although the cause of this poor healing rate is not well known, chronic degenerative lesions are most common in the elderly, and the elevated tension to which the suture is subjected makes determining the prognosis for healing decidedly more complicated [5–7]. Therefore, the first aim of this study was to develop an animal model to reproduce, as much as possible, the conditions observed in elderly patients who have rotator cuff tears. This model had to take into account key aspects such as age, the chronic nature of the injury and full-thickness injury to the SP tendon. Once the model was established, our second aim was to reproduce SP tendon detachment and delayed repair to mimic the repair of chronic rotator cuff tears in humans. We used three different repair strategies—sutures and type I collagen membranes with or without MSCs—to determine the most suitable treatment for the repair of chronic rotator cuff tears. Soslowsky’s group [9] have established rat models of chronic rotator cuff tears; however, the biomechanical properties of these models were not the same. Our rat model, in which we detached the SP tendon, is similar to that proposed by Soslowsky, but in contrast to the results for Soslowsky’s model, in the present study, the failure to load and stiffness progressively decreased with time postdetachment. This trend is most likely due to the erratic formation of scar tissue adhesions and their subsequent degradation over time.

Whereas most animal models have been developed in young animals, the current study used older animals to develop a chronic rotator cuff tear rat model in which the healing capacity is reduced, which might account for the lack of spontaneous healing. These results were confirmed by the presence of degenerative changes in the surrounding tissue, such as adipose tissue infiltrating the muscle, and mineral changes affecting the humeral head, which were observed macroscopically during dissection. Overall, our animal model showed decreased mechanical functioning as well as degenerative histological changes consistent with a chronic condition. The loss of strength as time progressed after treatment mimics the long-term high re-rupture rate observed in humans after treatment with primary surgery.

The ability of the MSCs to improve the biomechanical characteristics of the injured SP tendon compared with the other two strategies was encouraging. While the suture-treated animals showed a loss of biomechanical functioning as time progressed, in the MSC-membrane repair group, the maximum load to failure was statistically significantly improved at 3 months compared with suture groups. This finding indicates that the animals treated with MSCs have greater strength in the injured SP in terms of the resistance to breakage, which is consistent with the results of previous studies reporting significant increases in biomechanical functioning after implanting MSC collagen membranes in patellar tendon defects [18].

In summary, our study demonstrated that tendon treatment with MSCs significantly improved the tendon’s maximum load property and resulted in an augmented remodelled appearance compared with the other two methods at 3 months. It is possible that the healing process in the healthy state of the tendon is slowed, which could be explained by the animal model used in this study. First, to mimic the repair of chronic rotator cuff tears in humans, we began treatment in our animal model 1 month after detachment. It is known that healing is worse under these conditions than in acute injuries, although the reasons remain unclear. Second, MSCs were obtained from aged animals, which possess a decreased natural healing capacity. Although the exact mechanism responsible for the positive influence of MSCs on tendon healing remains uncertain, it is well known that MSCs have the capacity to differentiate into tenocytes, possess the capability to release growth factors and exhibit anti-inflammatory effects [19] at the site of injury, which could help provide a better healing environment and eventually lead to the regeneration of the damaged tissue.

Given the age of the rats used in the current study, the MSC differentiation rate might be slower, or these cells could even release growth factors in a slower manner than MSCs isolated from younger animals. We therefore believe that it is important to explore the effect of MSCs over a longer treatment period and to assess MSCs isolated from donors of various ages, especially in the case of aged animals.

In a rotator cuff tear model developed in young animals, Gulotta et al. [20] reported that there was a lack of differences with regard to the strength of the tendon and the alignment of the tendon collagen fibres when the animals were injected with MSCs compared with the vehicle-injected controls at 1 month, but these researchers neglected to evaluate the animals at later time points. However, they reported an improvement in the biomechanical and morphological properties in young animals at 45 days after the injection of MSCs.

Our findings suggest that the therapeutic use of allogeneic MSCs in type I collagen membranes for the repair of rotator cuff tears appears to be the most promising treatment in the long term. Nevertheless, questions concerning the optimal MSC dose, the effective time period, the optimal type of matrix material and donor age remain unanswered and will need to be addressed in future studies. This knowledge will hopefully lead to further advances that will improve the regeneration of injured SP tendons in the rotator cuff.

## Conclusions

Gross inspection of the specimens at the time of dissection revealed that the musculoskeletal unit was stiff, and the tendon appeared to be retracted. Although some studies using animal models have reported that the tendon heals naturally after release, producing a neotendon even in the absence of a surgical procedure using sutures, the aged rats used as our animal model retained the gap even at 3 months after the infliction of the lesion. The introduction of MSCs in the type I collagen membrane placed at the site of the surgically created defects resulted in the production of mechanically superior tissue, with improved SP tendon strength and stiffness at 3 months after treatment in comparison with the use of an acellular collagen membrane or sutures. More work is needed to determine the optimal matrix and dose of MSCs. The improvement is most likely influenced by mechanisms that affect the differentiation of MSCs and by factors that control the inflammatory environment at the site of injury. The application of MSCs to treat rotator cuff injuries may help improve the functionality of the shoulder. Future studies will be needed to test the effect of MSCs on tendon healing over a prolonged period of time.
